# Editorial Expression of Concern: New technique of end to side two layered and stented duct to mucosa pancreaticojejunostomy with omental wrapping during whipple operation

**DOI:** 10.1186/s12893-026-03686-6

**Published:** 2026-03-21

**Authors:** Hesham A. Elmeligy, Ahmed M. Azzam, Yousra Ossama, Mahmoud Rady

**Affiliations:** 1 General Surgery Department, Theodor Bilharz Research Institute (TBRI), Giza, Egypt; 2 Environmental Research Department, Theodor Bilharz Research Institute (TBRI, Giza, Egypt; 3 Pathology Department, October 6 University, Giza, Egypt

**Editorial Expression of Concern: BMC Surgery 25, 201 (2025)**

**https://doi.org/10.1186/s12893-025-02893-x**

In this article [1], Readers are alerted that concerns have been raised regarding the reliability of data presented in this article. Further editorial action will be taken if appropriate once the investigation into the concerns is complete and all parties have been given an opportunity to respond in full.
